# Hepatic Angiosarcoma Mimicking Sinusoidal Obstruction Syndrome: A Case Report

**DOI:** 10.7759/cureus.100234

**Published:** 2025-12-28

**Authors:** Margarida Mourato, Marta Machado, Rita Penaforte, Daniela R Silva, Miguel Achega

**Affiliations:** 1 Internal Medicine, Hospital Professor Doutor Fernando Fonseca, Amadora, PRT; 2 Infectious Diseases, Hospital Professor Doutor Fernando Fonseca, Amadora, PRT

**Keywords:** cholestatic liver injury, hepatic angiosarcoma, hepatic vascular tumor, sinusoidal obstruction syndrome, veno-occlusive disease

## Abstract

Hepatic angiosarcoma (HAS) is an uncommon malignant tumor that is often misdiagnosed because of its nonspecific presentation and overlapping imaging findings with benign vascular tumors. Sinusoidal obstruction syndrome (SOS) is a disorder of hepatic microcirculation, usually associated with myeloablative chemotherapy and hematopoietic stem cell transplantation. The clinical and pathological overlap between SOS and hepatic angiosarcoma may complicate the diagnosis of hepatic angiosarcoma, as illustrated by this case. We report the case of a sixty-year-old man who presented to the emergency department with progressive jaundice, abdominal discomfort, and weight loss exceeding 5% of his baseline. Laboratory workup revealed a predominantly cholestatic pattern, and serologies for viral, autoimmune, and metabolic liver diseases were negative. Also, there were no known risk factors for liver disease or SOS. Repeated imaging studies, including computed tomography (CT) and magnetic resonance imaging (MRI), revealed a large vascular hepatic lesion, suggestive of a benign lesion, and Doppler revealed findings compatible with SOS. Liver biopsy and hemodynamic studies indicated hepatic outflow obstruction. After clinical deterioration and expert multidisciplinary review of imaging and histology, malignant endothelial proliferation consistent with hepatic angiosarcoma was identified. Hepatic angiosarcoma can closely mimic SOS morphologically and hemodynamically. The absence of identifiable risk factors and the progressive evolution of hepatic changes should raise suspicion of malignancy, prompting multidisciplinary reassessment and careful histologic review.

## Introduction

Hepatic angiosarcoma (HAS) is a rare and aggressive malignant tumor with a tendency for vascular invasion, representing less than 2% of primary hepatic malignancies but accounting for many primary hepatic sarcomas [1]. Sinusoidal obstruction syndrome (SOS), previously known as veno-occlusive disease, results from endothelial sinusoidal injury and microvascular hepatic dysfunction with obstruction of small hepatic venules and post-sinusoidal portal hypertension. It mainly occurs after chemotherapy, bone marrow transplantation, or exposure to certain toxins [2]. 

Radiologically, hepatic angiosarcoma often exhibits a heterogeneous enhancement pattern on contrast-enhanced CT or MRI, but these findings may overlap significantly with those of benign vascular tumors such as cavernous hemangioma, particularly in early stages [3]. Angiosarcoma can even mimic the diffuse congestion and reticular enhancement pattern observed in SOS, making diagnosis particularly challenging and dependent on careful correlation between symptoms, imaging, and histopathological results [4,5]. Distinguishing between these entities is essential because the therapeutic and prognostic implications differ markedly, given that angiosarcomas are highly aggressive with poor outcomes if not promptly recognized [6].

This case highlights how hepatic angiosarcoma can imitate both benign hepatic vascular lesions and SOS, underscoring the importance of multidisciplinary diagnostic integration of clinical findings, imaging, hemodynamic, and histopathological results.

## Case presentation

A 60-year-old man with type 2 diabetes mellitus and b-thalassemia minor presented to the emergency department of a peripheral-care hospital with progressive jaundice, fatigue, and mild abdominal distension evolving over two months, associated with approximately 6% unintentional weight loss. He denied alcohol consumption, occupational exposure to toxins, or hepatotoxic medication intake, including paracetamol, herbal products, or anabolic steroids.

Upon examination, he appeared slightly jaundiced, and on abdominal palpation, there was right upper quadrant tenderness; no other findings were relevant. Laboratory workup revealed a predominantly cholestatic pattern (R factor 1.4), aspartate aminotransferase (AST) 60 U/L, alanine aminotransferase (ALT) 69 U/L, alkaline phosphatase (ALP) 149.88 U/L, and gamma-glutamyl transferase (GGT) 389 U/L, as well as total bilirubin of 4.08 mg/dL (direct 3.20 mg/dL). Albumin was 3.1 g/dL, and the international normalized ratio (INR) was 1.3. Serologies for viral hepatitis A, B, C, and E viruses, Epstein-Barr virus, cytomegalovirus, and autoimmune liver disease were negative. Metabolic studies, including a-1-antitrypsin, ceruloplasmin, and hemochromatosis mutation testing, were normal. Alpha-fetoprotein was 1.4 ng/mL.

Despite supportive therapy, and while we awaited the results of the complementary diagnostic tests that had been requested, the patient’s liver function continued to deteriorate within the following weeks. Table 1 summarizes the evolution of laboratory parameters, showing progressive increases in bilirubin and alkaline phosphatase, accompanied by mild coagulopathy and decreased albumin levels. 

**Table 1:** Laboratory test results at admission, day 10, and day 50 are shown alongside local reference ranges, demonstrating progressive cholestasis and mild coagulopathy. AST: aspartate aminotransferase; ALT: alanine aminotransferase; ALP: alkaline phosphatase; GGT: gamma-glutamyl transferase; LDH: lactate dehydrogenase; INR: international normalized ratio; MCV: mean corpuscular volume

Laboratory parameter	Admission	Day 10	Day 50	Reference range
AST (U/L)	60	114	223	< 40
ALT (U/L)	69	107	138	< 41
ALP (U/L)	149.88	167.12	210.23	40.0 – 130.0
GGT (U/L)	389	598	556	< 60
Total bilirubin (mg/dL)	4.08	4.13	9.24	< 1.20
Direct bilirubin (mg/dL)	3.20	2.28	6.46	< 0.20
Albumin (g/dL)	3.1	2.8	2.8	3.5 – 5.0
INR	1.3	1.5	1.6	< 1.2
LDH (U/L)	231	260	327	135 - 125
Hemoglobin (g/dL)	9.1	8.0	7.1	13.0 – 17.0
MCV (fL)	58.5	67.0	65.8	83 - 101

Abdominal Doppler ultrasound revealed mild ascites, a well-defined predominantly hyperechoic lesion in the right hepatic lobe, measuring approximately 7 cm, with internal vascularity and peripheral nodular flow. The portal venous flow was preserved, hepatic venous waveforms were monophasic, and the hepatic arterial resistive index was elevated (0.76). These findings were found to be consistent with a benign vascular lesion, most likely a cavernous hemangioma, and with sinusoidal congestion and SOS, as stated in Wiland et al.'s report [7]. These features reflect increased sinusoidal resistance and impaired hepatic venous compliance, with loss of the normal triphasic venous pattern and compensatory elevation of hepatic arterial resistances, changes typically seen in SOS [2,4,5,8].

**Figure 1:**
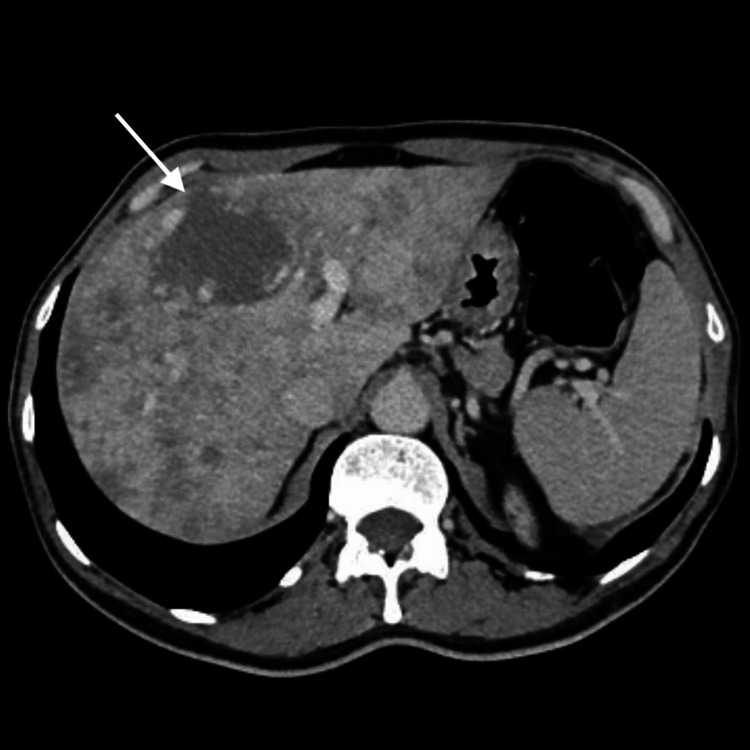
Contrast-enhanced CT (axial view) of the abdomen showing the liver with globular morphology and diffusely heterogenous parenchyma in both arterial and venous phases due to the presence of multiple scatteres hypoenhancing areas without nodular morphology. Heterogeneity decreases in the delayed phase, which is suggestive of perfusion alterations. Also, a nodular lesion is visible at the transition between segment VIII and IV-A with 60mm with peripheral nodular enhancement and centripetal fill-in suggestive of a hemangioma.

Triphasic CT imaging (Figure 1) revealed diffuse hepatic heterogeneity with a 6 cm lesion concerning the junction of segments IV and VIII, with peripheral nodular enhancement and features suggestive of a benign vascular lesion, most likely a hemangioma. 

**Figure 2:**
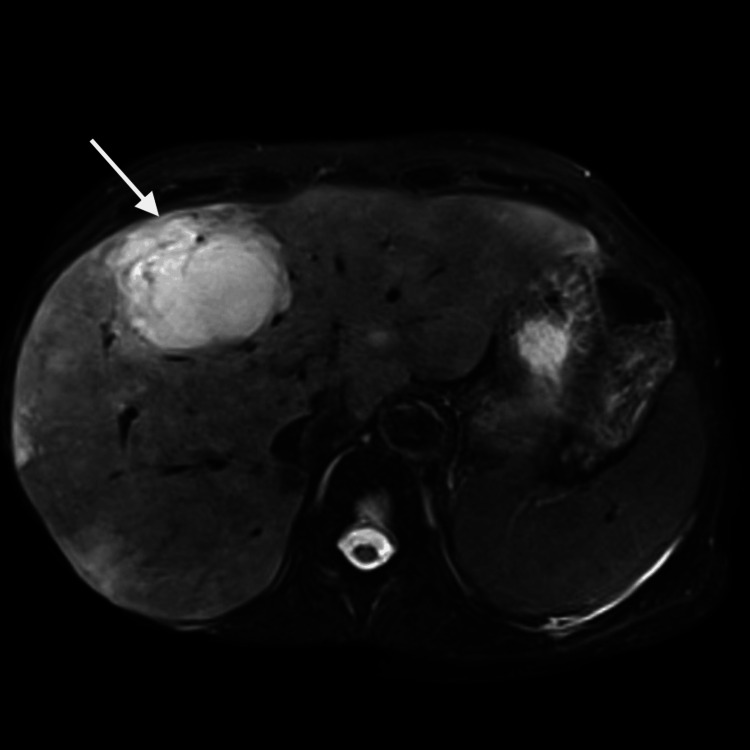
Axial T2-weighted MRI of the upper abdomen demonstrating hepatomegaly, approximately 18.2 cm in longitudinal diameter, with lobulated contours and markedly heterogenous signal intensity dysplaing a mottled appearance and several ill-defined hypoenchancing foci. A thin, filiform filling defect is seen in the central lumen of the right hepatic vein, along with significant narrowing of the retrohepatic inferior vena cava at its uppermost segment.

An abdominal MRI was performed (Figure 2), which confirmed hepatomegaly (18.2 cm) and a 77 × 53 mm hyper-vascular lesion in segment IV, with mottled enhancement and heterogeneous parenchymal signal, initially interpreted as compatible with a hepatic hemangioma and sinusoidal obstruction or venous outflow impairment. The findings were highly suggestive of veno-occlusive disease of indeterminate etiology. The findings were highly suggestive of veno-occlusive disease of indeterminate etiology. 

During hospitalization, the patient’s cholestasis and jaundice worsened, as illustrated in Table 1. However, the patient remained clinically stable, with mild ascites, which was insufficient for paracentesis, and with no evidence of encephalopathy or other manifestations of advanced liver dysfunction.

A percutaneous liver biopsy was performed on hospital day 16, and microscopic examination revealed marked sinusoidal dilatation, congestion, and mild pericellular fibrosis without significant inflammation or atypical cells. Hepatocytes showed atrophy and focal cholestasis adjacent to dilated vascular spaces. These findings were initially interpreted as suggestive of vascular outflow obstruction or SOS.

Given the concomitant and worsening anemia, despite a previous diagnosis of β-thalassemia, and, in order to rule out any possible risk factor for SOS, a bone marrow aspirate was performed on hospital day 18. It showed a hypercellular marrow with marked erythroid hyperplasia, dyserythropoiesis, and markedly increased storage iron without sideroblastic iron or malignant infiltration. Occasional hemophagocytes were seen, consistent with reactive changes secondary to β-thalassemia and chronic liver disease.

**Figure 3:**
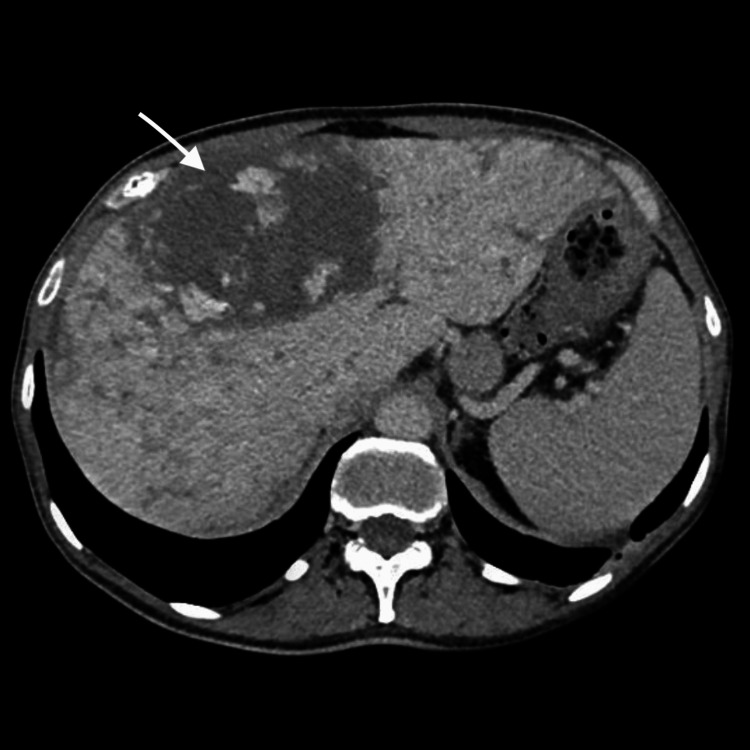
Axial contrast-enhanced CT scan of the abdomen in the portal venous phase (Hospital day 45). Moderate hepatomegaly with lobulated contours and diffusely heterogenous structure with a diffuse mottled appearance continues to be seen. Multiple nodular foci of various sizes can be seen, corresponding to previously known hemangiomas, the largest measuring approximately 86 x 75 mm in segment IV.

On the 20th day of hospitalization, the patient experienced an acute upper airway obstruction secondary to food aspiration, which progressed to cardiac arrest. He was promptly resuscitated and admitted to the intensive care unit, where he remained for three weeks due to difficulty in ventilator weaning, delaying the continuation of diagnostic investigations. After stabilization, the patient returned to the internal medicine ward, and follow-up CT imaging showed rapid enlargement of the dominant hepatic lesion to 86 x 75 mm with central necrosis, with irregular peripheral enhancement and progressive centripetal filling, with ill-defined margins and heterogeneous parenchymal enhancement of the surrounding liver (Figure 3). There was associated segmental capsular retraction and increasing heterogeneity of the remaining hepatic parenchyma, findings that were atypical for benign vascular lesions. The portal and hepatic veins remained patent with no biliary dilation or extrahepatic disease identified. 

Given the persistent cholestasis without a clear etiology and the suspicion of SOS, a transjugular liver biopsy with hemodynamic assessment was performed on hospital day 39, and confirmed elevated hepatic venous pressures, indicating post-sinusoidal resistance. The procedure demonstrated a free hepatic venous pressure (FHVP) of 6 mmHg, a wedged hepatic venous pressure (WHVP) of 18 mmHg, resulting in a hepatic venous pressure gradient (HVPG) of 12 mmHg. The normal FHVP excluded prehepatic or cardiac causes, while the elevated WHVP and HVPG indicated intrahepatic sinusoidal resistance, findings consistent with the presumptive diagnosis of SOS. Also, microscopic examination was similar to the initial biopsy performed and revealed no cellular atypia or inflammatory infiltrate. 

The case was reviewed in a multidisciplinary meeting, since these changes raised concern for a primary malignant vascular process, and, given the atypical evolution and absence of known risk factors, a full histological re-evaluation was performed. Expert pathology review identified malignant endothelial proliferation forming irregular anastomosing vascular channels that dissected between hepatic plates and replaced the normal lobular architecture. Some areas exhibited epithelioid morphology with solid nests of atypical endothelial cells, whereas others showed spindle-cell features within fibrotic stroma. The neoplastic endothelial cells displayed enlarged, hyperchromatic, and pleomorphic nuclei, occasional mitotic figures, and focal multilayering along vascular spaces. Immunohistochemistry was positive for CD31, CD34, p53, and MYC, with Ki-67 > 10%, confirming primary hepatic angiosarcoma.

The patient was referred to a specialized center; however, his condition continued to deteriorate with worsening cholestasis and hepatic dysfunction. Furthermore, given the multifocal and infiltrative nature of the disease, curative treatment was deemed unfeasible, and palliative management was pursued.

## Discussion

Hepatic angiosarcoma is a rare and highly aggressive malignant vascular tumor, often presenting with nonspecific clinical and biochemical findings [1]. Because early imaging may show features resembling benign hepatic lesions, diagnosis is frequently delayed or missed [3]. In this case, the initial CT, the MRI, and abdominal Doppler ultrasound reports described the hepatic lesion as well-defined and vascular, suggesting a possible cavernous hemangioma, while the associated hemodynamic findings were interpreted as consistent with SOS. These interpretations led to diagnostic uncertainty and consideration of non-neoplastic causes such as toxic, vascular, or inflammatory liver injury. SOS and hepatic angiosarcoma share overlapping pathophysiologic mechanisms of sinusoidal endothelial injury and congestion, which may explain this radiologic similarity [2,4,5].

The Doppler ultrasound and transjugular hemodynamic findings in this patient were both compatible with SOS, showing monophasic hepatic venous waveforms, increased hepatic arterial resistive index, and a markedly elevated hepatic venous pressure gradient (HVPG 12 mmHg), with a normal free hepatic venous pressure. These findings reflected sinusoidal or post-sinusoidal resistance and were therefore interpreted as consistent with SOS at the time [4,5,9]. However, the absence of known risk factors for endothelial injury and the progressive radiologic evolution of the hepatic lesions prompted reconsideration of the diagnosis. 

Histopathological and immunohistochemical reevaluation were essential in establishing the diagnosis. The microscopic features of irregular, anastomosing vascular channels lined by atypical endothelial cells with nuclear pleomorphism and mitoses confirmed the malignant nature of the lesion. The diffuse positivity for endothelial markers CD31, CD34, ERG, and FLI-1 confirmed vascular differentiation, while nuclear expression of p53 and MYC supported malignant transformation. The elevated proliferation index (Ki-67 15-20%) further indicated high proliferative activity. These findings reinforced the diagnosis of primary hepatic angiosarcoma [10-12].

Hepatic angiosarcoma can therefore imitate both the imaging and hemodynamic profile of SOS, as well as the enhancement patterns of benign vascular tumors such as hemangioma. In some reported cases, angiosarcoma has initially presented with histologic features suggestive of SOS, later reclassified upon progression and re-evaluation [6,7,13]. This highlights the need for continuous multidisciplinary review when clinical or imaging evolution contradicts a benign diagnosis.

The prognosis of hepatic angiosarcoma remains dismal, with a median survival of under one year in most series [1,14]. Surgical resection offers the best chance for prolonged survival, but only a minority of patients present with resectable disease [15]. Systemic therapy yields limited benefit, and liver transplantation is contraindicated due to high recurrence risk [16,17]. Additionally, contemporary overviews reinforce the extremely poor prognosis and high diagnostic delay of hepatic angiosarcoma [18]. Early multidisciplinary reassessment, including expert pathology review, remains critical for timely diagnosis and appropriate management.

## Conclusions

This case illustrates the diagnostic challenge posed by hepatic angiosarcoma, which can mimic benign vascular lesions and SOS on imaging studies and occasionally on histology as well, contributing to diagnostic delay. The case underscores the importance of integrating clinical, imaging, hemodynamic, and histopathologic findings, as well as the critical role of multidisciplinary assessment in atypical or progressive cases to avoid delayed recognition. 

Given the aggressive nature and poor prognosis of these types of tumours, prompt identification is essential, yet often difficult, especially when imaging and biopsy suggest benign lesions or other hepatic disorders. This case reinforces how evolving clinical course, rapid lesion growth, and discordant findings should encourage renewed diagnostic scrutiny, including pathological review. Ultimately, earlier recognition may expand treatment options for selected patients; therefore, it is important to maintain a high index of suspicion in complex hepatic presentations where vascular or infiltrative malignancy cannot be confidently excluded.

## References

[1] Locker GY, Doroshow JH, Zwelling LA, Chabner BA (1979). The clinical features of hepatic angiosarcoma: a report of four cases and a review of the English literature. Medicine (Baltimore).

[2] DeLeve LD, Shulman HM, McDonald GB (2002). Toxic injury to hepatic sinusoids: sinusoidal obstruction syndrome (veno-occlusive disease). Semin Liver Dis.

[3] Yasir S, Torbenson MS (2019). Angiosarcoma of the liver: clinicopathologic features and morphologic patterns. Am J Surg Pathol.

[4] Simpson S, Breshears E, Basavalingu D (2024). Review of imaging findings in hepatic veno-occlusive disease. Eur J Radiol.

[5] Dignan FL, Wynn RF, Hadzic N (2013). BCSH/BSBMT guideline: diagnosis and management of veno-occlusive disease (sinusoidal obstruction syndrome) following haematopoietic stem cell transplantation. Br J Haematol.

[6] Ha FS, Liu H, Han T, Song DZ (2022). Primary hepatic angiosarcoma manifesting as hepatic sinusoidal obstruction syndrome: a case report. World J Gastrointest Oncol.

[7] Wiland HO 4th, Pai RK, Purysko AS (2012). Hepatic angiosarcoma mimicking sinusoidal obstruction syndrome/venoocclusive disease: a pathologic-radiologic correlation. Ann Diagn Pathol.

[8] Fan CQ, Crawford JM (2014). Sinusoidal obstruction syndrome (hepatic veno-occlusive disease). J Clin Exp Hepatol.

[9] Hosoki T, Kuroda C, Tokunaga K, Marukawa T, Masuike M, Kozuka T (1989). Hepatic venous outflow obstruction: evaluation with pulsed duplex sonography. Radiology.

[10] Young RJ, Brown NJ, Reed MW, Hughes D, Woll PJ (2010). Angiosarcoma. Lancet Oncol.

[11] Flabouris K, McKeen S, Chaves Gomes D, Chaudhuri D, Russell P (2021). Hepatic angiosarcoma: pitfalls in establishing a diagnosis. SAGE Open Med Case Rep.

[12] Hogeboom-Gimeno AG, van Ravensteijn SG, Desar IM (2023). MYC amplification in angiosarcoma depends on etiological/clinical subgroups - diagnostic and prognostic value. Ann Diagn Pathol.

[13] Jiang L, Xie L, Li G, Xie H, Fang Z, Cai X, Chen Y (2021). Clinical characteristics and surgical treatments of primary hepatic angiosarcoma. BMC Gastroenterol.

[14] Roohani S, Rotermund T, Ehret F (2024). Angiosarcoma: clinical outcomes and prognostic factors, a single-center analysis. J Cancer Res Clin Oncol.

[15] Cao J, Wang J, He C, Fang M (2019). Angiosarcoma: a review of diagnosis and current treatment. Am J Cancer Res.

[16] Zheng YW, Zhang XW, Zhang JL, Hui ZZ, Du WJ, Li RM, Ren XB (2014). Primary hepatic angiosarcoma and potential treatment options. J Gastroenterol Hepatol.

[17] Tran Minh M, Mazzola A, Perdigao F, Charlotte F, Rousseau G, Conti F (2018). Primary hepatic angiosarcoma and liver transplantation: radiological, surgical, histological findings and clinical outcome. Clin Res Hepatol Gastroenterol.

[18] Chaudhary P, Bhadana U, Singh RA, Ahuja A (2015). Primary hepatic angiosarcoma. Eur J Surg Oncol.

